# Saprophytic *Bacillus* Accelerates the Release of Effective Components in Agarwood by Degrading Cellulose

**DOI:** 10.3390/molecules27041428

**Published:** 2022-02-20

**Authors:** Huizhen Yang, Runying He, Yao Cui, Ying Li, Xizhen Ge

**Affiliations:** College of Biochemical Engineering, Beijing Union University, Beijing 100023, China; yanghuizhen0228@163.com (H.Y.); herunying716@163.com (R.H.); cy019157@163.com (Y.C.)

**Keywords:** saprophytic *Bacillus*, Agarwood, cellulose, effective components

## Abstract

The value of Agarwood increases with time due to the gradual release of its major components, but the mechanism behind this remains unclear. Herein we reveal that the potential driving force of this process is the degradation of cellulose in Agarwood by its saprophytic *Bacillus* *subtilis*. We selected 10-year-old Agarwood from different places and then isolated the saprophytic bacteria. We confirmed these bacteria from different sources are all *Bacillus* and confirmed they can degrade cellulose, and the highest cellulase activity reached 0.22 U/mL. By co-cultivation of the bacterium and Agarwood powder, we found that three of the strains could release the effective components of Agarwood, while they had little effect in increasing the same components in living *Aquilaria sinensis*. Finally, we demonstrated that these saprophytic *Bacillus* *subtilis* have similar effects on *Zanthoxylum bungeanum* Maxim and *Dalbergiaod orifera* T. Chen, but not on *Illicium verum* Hook. f, *Cinnamomum cassia* Presl and *Phellodendron chinense* Schneid. In conclusion, our experiment revealed that the saprophytic *Bacillus* release the effective components of Agarwood by degrading cellulose, and we provide a promising way to accelerate this process by using this bacterial agent.

## 1. Introduction

Agarwood, the resinous portions of *Aquilaria sinensis*, is widely used as natural fragrance or precious medicine in China, Japan, India and other Southeast Asian countries [1]. After being wounded by external forces, *Aquilaria sinensis* is infested by insects, bacteria or fungi, and finally forms a secretion near the wound, accumulating to form Agarwood over time [1]. The major active components of Agarwood have been identified as 2-(2-phenylethyl) chromone, simple phenolic compounds, triterpenoids and sesquiterpenes [2]. The value of Agarwood is strongly related to its storage time, and the aged Agarwood is of much higher value [1]. However, the reason for the increased value of Agarwood with storage time remains unknown.

Plant endophytes are microorganisms that exist in a specific period or the entire life history of a plant. These microorganisms, which are mainly archaea [3], bacteria [4] or fungi [5], usually form harmonious relationship with the host. Beneficial endophytes form a mutually symbiotic relationship with plants during their growth [6,7], which enhance the stress resistance of plants [8] and are used for biological control [9,10,11]. It has been demonstrated that endophytic bacteria in medicinal plants can produce a variety of bioactive compounds, which are promising in medical, agricultural and industrial applications [12]. At the same time, endophytic bacteria show significant plant growth-promoting activities in vitro, including auxin synthesis, diazotrophy, phosphate solubilization, siderophores, and lytic enzyme (chitinase, cellulase, protease, and lipase) production [13]. Previous studies on endophytic bacteria of Agarwood mainly focused on isolation and identification of endophytic microorganisms [14,15], composition and distribution of endophytic bacteria colony structure [16,17,18], and secondary metabolites of endophytic fungi [19,20]. These microorganisms displayed potential in increasing the major components of *Aquilaria sinensis* [19,20]. However, although these microorganisms are suggested to be active in live *Aquilaria sinensis,* still little is known about their roles in Agarwood. We suspect these fungi or bacteria, although rarely reported, are still alive as saprophytic microorganisms in Agarwood and have an undiscovered role in releasing the effective components of Agarwood.

In this study, we aimed to investigate the role of saprophytic bacteria in increasing the release of the components of Agarwood. We selected 10-year-old Agarwood and isolated the saprophytic bacteria. We obtained seven *Bacillus subtilis* strains and then confirmed they are beneficial to the release of Agarwood’s active ingredients by degrading cellulose. We demonstrated that these bacteria had little effect on *Aquilaria sinensis* while functioning in Agarwood. We revealed a novel role of these saprophytic bacteria in increasing the value of Agarwood.

## 2. Results

### 2.1. Isolation and Characterization of Saprophytic Bacteria from 10-Year-Old Agarwood

**Figure 1:**
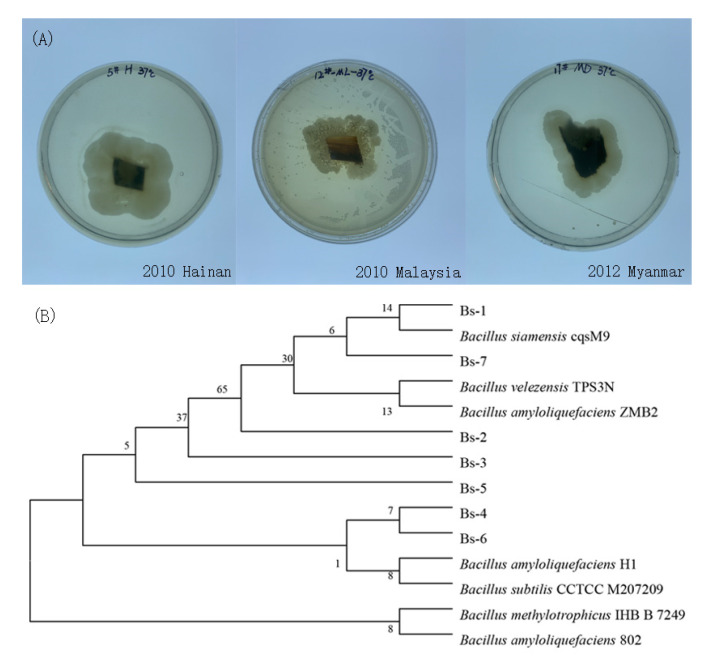
Isolation and identification of Agarwood saprophytic bacteria. (**A**): 10-year-old Agarwood from different places was washed by ethanol followed by drying in a clean biosafety hood. Then a small piece was cut from the center of each sample and cultivated in a PDA medium. Strains grown around the small slides indicated they were saprophytic microorganisms. (**B**): Phylogenetic tree constructed based on the alignment of 16S rDNA sequences.

**Figure A1:**
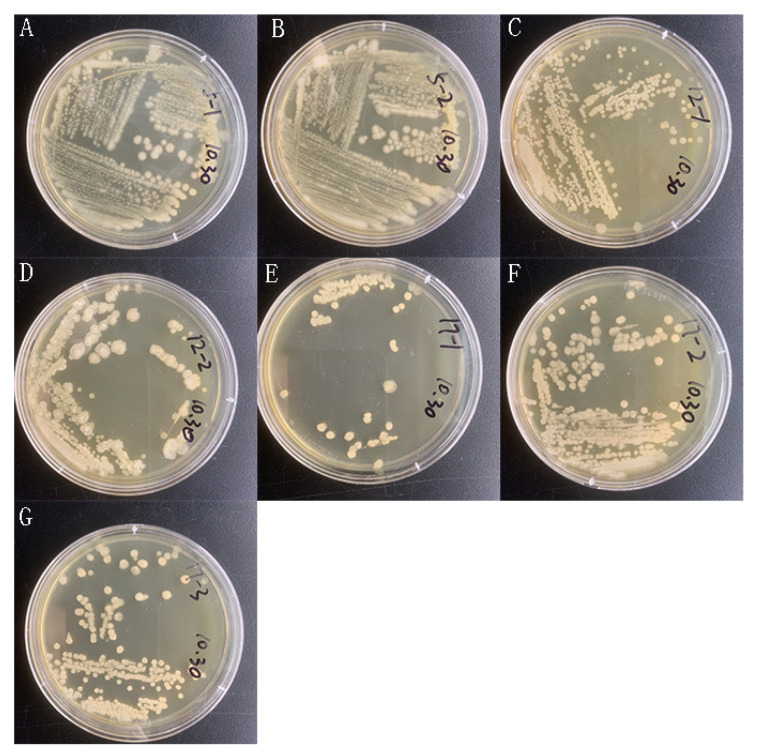
Isolation of agarwood saprophytic bacteria. (**A–G**): Bacterial suspension of Bs-1 to Bs-7 were dipped and streaked on the PDA medium. Then the medium was cultured at 37 °C for 12 h.

Aged Agarwood is often more favored by consumers due to the accumulation of resin and the release of fragment components [1]. We suspect that the saprophytic endophytes have a certain effect on Agarwood, so we selected 10-year-old Agarwood from Hainan, Malaysia, and Myanmar to isolate their saprophytic microorganisms (Figure 1, Figure A1). After initial assessment from the shape of the colony, we deduced that these were saprophytic bacteria. Then, 16S rDNA sequencing was conducted. The results indicated that these bacteria were *Bacilli*, and they were named as Bs-1 to Bs-7. By comparing to the NCBI database, we constructed a phylogenetic tree with the highest credible strains. The results indicated that, apart from Bs-1, these strains had low genetic relationship with sequenced *Bacillus* isolates, suggesting these bacteria may not have been discovered before.

### 2.2. Two of the Saprophytic Bacillus Promotes the Release of Major Components of Agarwood

**Figure 2:**
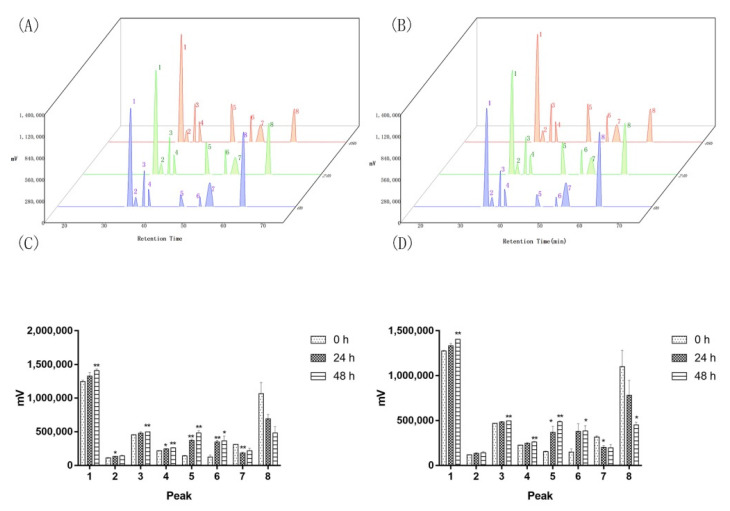
Agarwood powder fermentation (**A**,**C**): Bs-2 was added in Agarwood powder and sterilized saline, and then the suspension was thoroughly mixed and incubated. Samples were taken at 0 h, 24 h, and 48 h and filtered for HPLC analysis. (**B**,**D**): Bs-4 was added to Agarwood powder and sterilized saline, and then the suspension was thoroughly mixed and incubated. Samples were taken at 0 h, 24 h, and 48 h and filtered for HPLC analysis. Values are expressed as mean ± SEM, * *p* < 0.05 and ** *p* < 0.001 vs. control.

The *Bacillus* strains were isolated from 10-year-old Agarwood, implying they can survive for long time in Agarwood. In order to explore their exact role in Agarwood, we designed a medium with Agarwood powder and saline, and selected the faster-growing Agarwood endophytes Bs-2 and Bs- 4 to carry out fermentation (Figure 2). Surprisingly, the results indicated that as the fermentation time prolonged, the major components of Agarwood were gradually released into the medium. Among them, the peak of agarotetrol (peak 1) increased significantly, and others (peak 1, Peak 3, peak 4, peak 5, peak 6) were also significantly increased with the treatment of the two *Bacillus*, while no changes were found without bacteria added.

### 2.3. Saprophytic Bacilli Are Cellulase-Producing Strains

**Figure 3:**
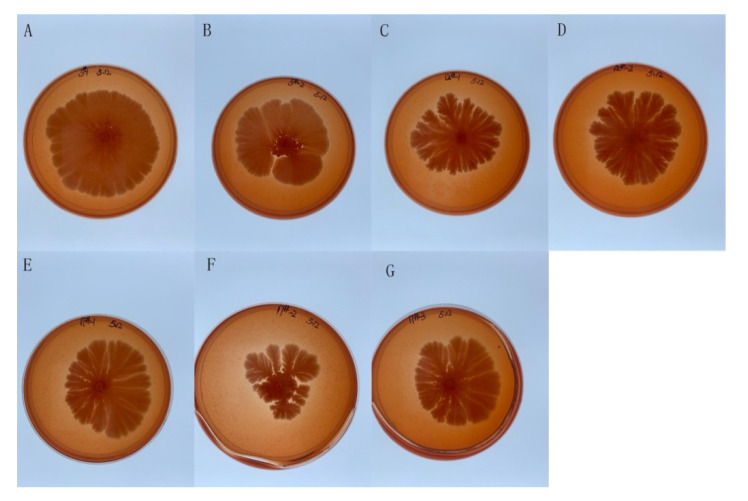
Saprophytic *Bacilli* are cellulase-producing strains. (**A**–**G**): Bacterial suspension of Bs-1 to Bs-7 were dipped and placed on the center of CMC-Congo red medium. Then the medium was cultured at 37 °C for 48 h.

Since saprophytic *Bacilli* of Agarwood can release the major components of the Agarwood, we speculate that these saprophytic *Bacilli* can use the cellulose in the Agarwood as a carbon source for survival. Therefore, we conducted experiments to detect the cellulase activity of these bacteria. We used both the transparent circle experiment [21] (Figure 3) and the filter paper enzyme activity experiment [22] (Table 1) to test the cellulase-producing ability of these strains. After culturing Bs-1 to Bs-7 on CMC-Congo red medium for two days, clear circles were found near the colonies, indicating that the seven strains have the ability to produce cellulase. In parallel, the filter paper enzyme activity of these strains was determined and the results indicated that the strains had different abilities to degrade cellulose. Among them, the strains Bs-2 and Bs-4 showed the highest enzyme activities.

The enzyme activities of the seven strains were significantly different from the blank.

### 2.4. Saprophytic Bacillus Had a Limited Effect on Degrading Major Effective Components of Agarwood

**Figure 4:**
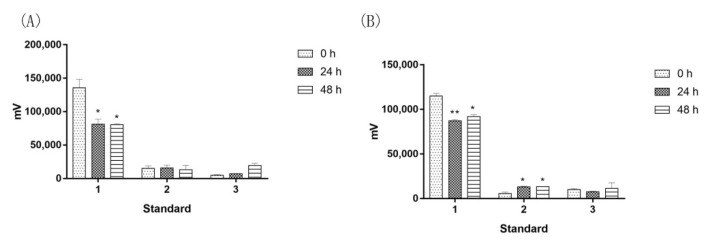
Effect of saprophytic *Bacilli* on major effective components of Agarwood (**A**): Bs-2 was separately put in a test tube with liquid LB medium and different major standards (Standard 1: agarotetrol; Standard 2: 6,7-dimethoxy-2-(2-phenylethyl)chromone; Standard 3: 2-(2-phenylethyl)chromone), and then the mixture was incubated. Samples were taken at 0 h, 24 h and 48 h, and then filtered for HPLC analysis. (**B**): Bs-4 was separately put in a test tube with liquid LB medium and different major standards (Standard 1: agarotetrol; Standard 2: 6,7-dimethoxy-2-(2-phenylethyl)chromone; Standard 3: 2-(2-phenylethyl)chromone), and then the mixture was incubated. Samples were taken at 0 h, 24 h and 48 h, and then filtered for HPLC analysis. Values are expressed as mean ± SEM, * *p* < 0.05, and ** *p* < 0.001 and vs. control.

The above data indicated that Agarwood saprophytic endophytes can degrade cellulose, and it is also important to determine whether they can assume the major components of Agarwood. We selected three standards reported in the literature, agarotetrol, 6,7-dimethoxy-2-(2-phenylethyl)chromone and 2-(2-phenylethyl)chromone [23,24] (Figure 4), and we used Bs-2 and Bs-4 to test whether they can digest these components. The results demonstrated that when Bs-2 is used to act on the standards, agarotetrol is partially consumed at 0 h to 24 h, while the titer was kept from 24 h to 48 h. Another two components, 6,7-dimethoxy-2-(2-phenylethyl) chromone and 2-(2-phenylethyl) chromone, were not digested by these strains. Similar to Bs-2, Bs-4 partially degraded agarotetrol, while it had no effect on the other components. However, overall, Agarwood powder fermentation still increased the titer of agarotetrol, indicating that the release effect of endophytic bacteria on agarotetrol is faster than the decomposition effect.

### 2.5. Common Chinese Herbal Medicine Powder Fermentation

**Figure 5:**
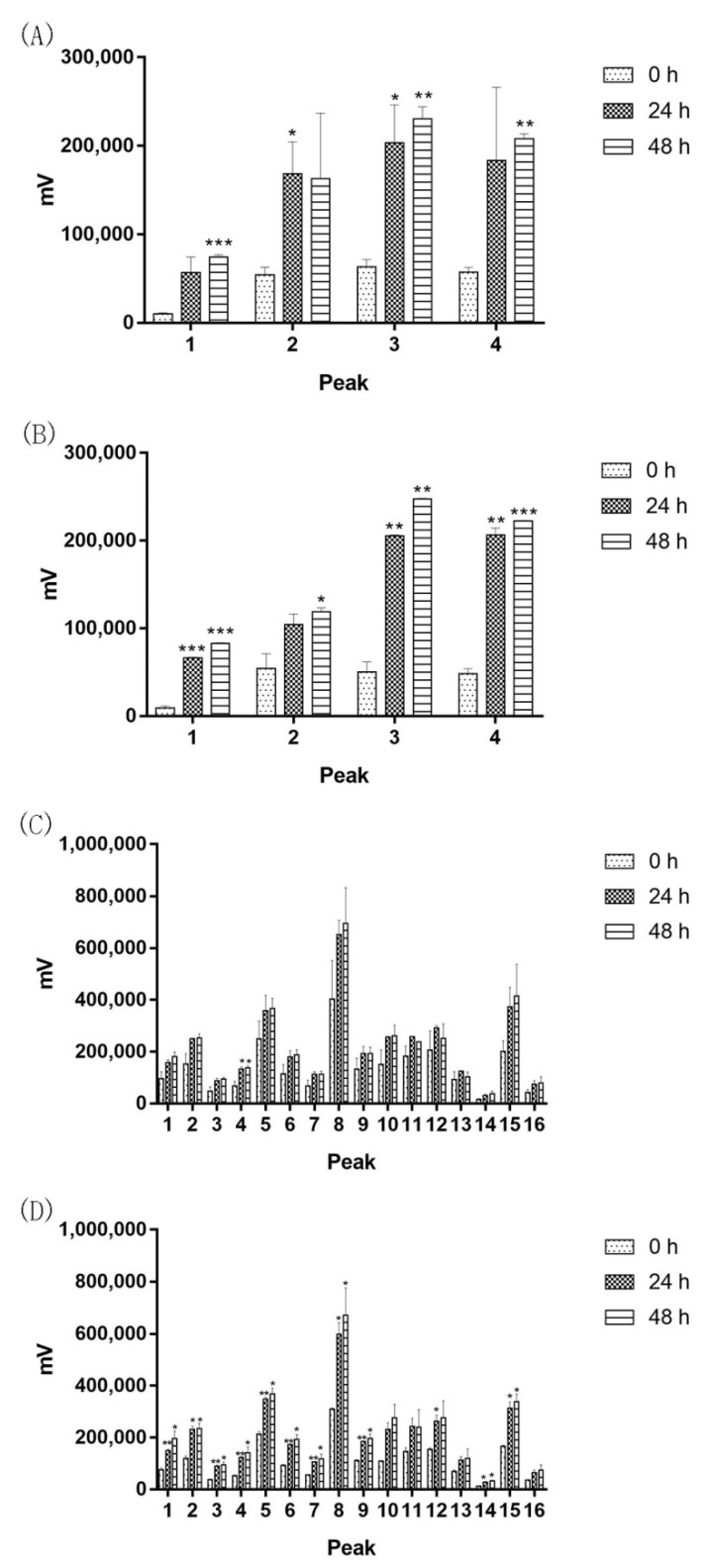
Effect of saprophytic *Bacilli* on *Dalbergiaod orifera* T. Chen and *Zanthoxylum bungeanum* Maxim. (**A**,**B**): Bs-2 and Bs-4 were separately put in test tubes with liquid LB medium and *Dalbergiaod orifera* T. Chenpowder, and then the mixture was incubated. Samples were taken at 0 h, 24 h and 48 h, and then filtered for HPLC analysis. (**C**,**D**): Bs-2 and Bs-4 were separately put in test tubes with liquid LB medium and *Zanthoxylum bungeanum* Maxim powder, and then the mixture was incubated. Samples were taken at 0 h, 24 h and 48 h, and then filtered for HPLC analysis. Values are expressed as mean ± SEM, * *p* < 0.05, ** *p* < 0.001, and *** *p* < 0.0001 vs. control.

**Figure A2:**
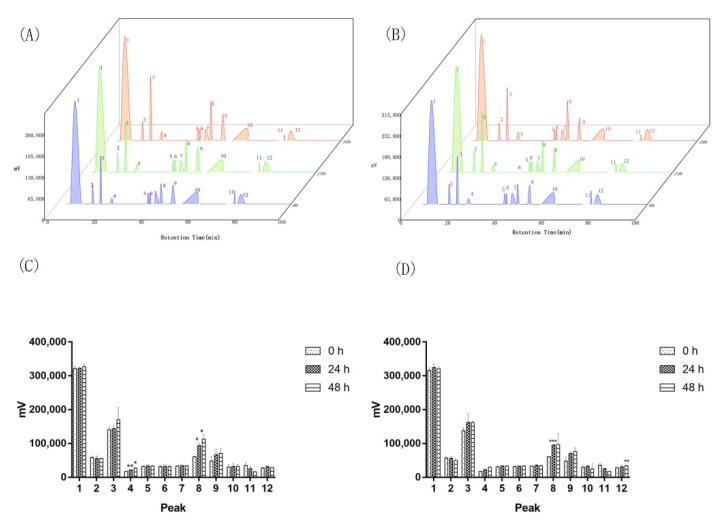
*Illicium verum* Hook. f powder fermentation (**A**,**C**): Bs-2 was added in Anise powder and sterilized saline, and then the suspension was thoroughly mixed and incubated. Samples were taken at 0 h, 24 h, and 48 h and filtered for HPLC analysis. (**B**,**D**): Bs-4 was added in Anise powder and sterilized saline, and then the suspension was thoroughly mixed and incubated. Samples were taken at 0 h, 24 h, and 48 h and filtered for HPLC analysis. * *p* < 0.05, ** *p* < 0.001, and *** *p* < 0.0001.

**Figure A3:**
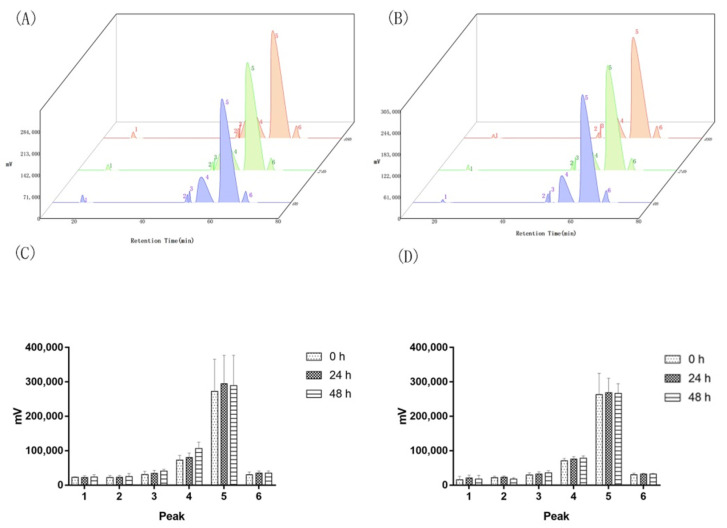
*Cinnamomum cassia* Presl powder fermentation (**A**,**C**): Bs-2 was added in Cassia powder and sterilized saline, and then the suspension was thoroughly mixed and incubated. Samples were taken at 0 h, 24 h, and 48 h and filtered for HPLC analysis. (**B**,**D**): Bs-4 was added in Cassia powder and sterilized saline, and then the suspension was thoroughly mixed and incubated. Samples were taken at 0 h, 24 h, and 48 h and filtered for HPLC analysis.

**Figure A4:**
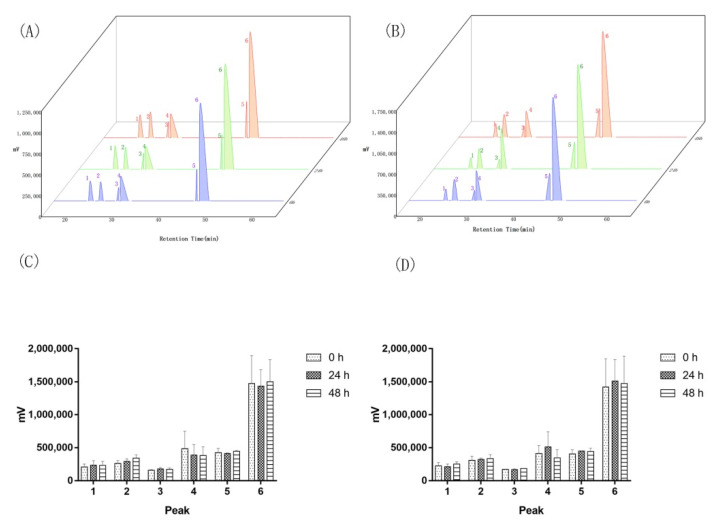
*Phellodendron chinense* Schneid powder fermentation (**A**,**C**): Bs-2 was added in Golden cypress powder and sterilized saline, and then the suspension was thoroughly mixed and incubated. Samples were taken at 0 h, 24 h, and 48 h and filtered for HPLC analysis. (**B**,**D**): Bs-4 was added in Golden cypress powder and sterilized saline, and then the suspension was thoroughly mixed and incubated. Samples were taken at 0 h, 24 h, and 48 h and filtered for HPLC analysis.

We confirmed that Agarwood saprophytic endophytes did indeed degrade cellulose and release the major components of Agarwood. In order to identify whether there was a broad effect or not, we selected *Dalbergiaod orifera* T. Chen, *Illicium verum* Hook. f, *Cinnamomum cassia* Presl and *Phellodendron chinense* Schneid, *Zanthoxylum bungeanum* Maxim for powder fermentation experiments (Figure 5, Figure A2, Figure A3, Figure A4). The results indicated that these strains had similar effects on *Zanthoxylum bungeanum* Maximand *Dalbergiaod orifera* T. Chen. The components of *Dalbergiaod orifera* T. Chen 4 (peak 1–peak 4) increased significantly. Except for compounds 11, 12, and 13, all other compounds (peak 1–peak 10, peak 14–peak 16) of *Zanthoxylum bungeanum* Maxim increased significantly. In contrast, no similar effect was observed toward *Illicium verum* Hook. f, *Cinnamomum cassia* Presl and *Phellodendron chinense* Schneid.

### 2.6. Saprophytic Bacilli Had a Limited Effect in Aquilaria sinensis

**Figure 6:**
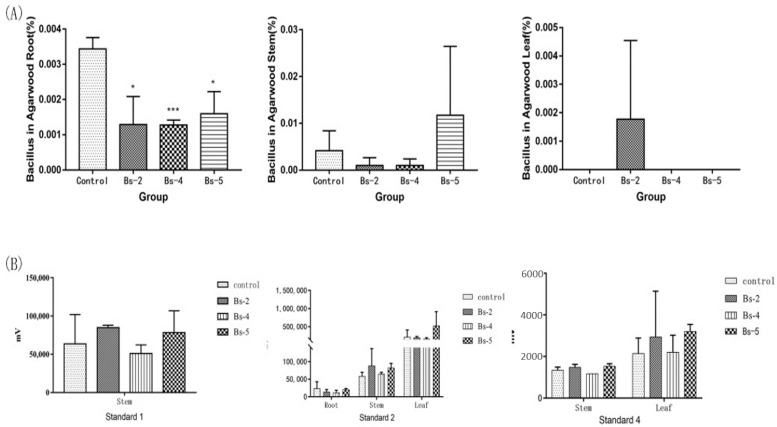
Effect of saprophytic *Bacilli* on *Aquilaria sinensis*. (**A**): The suspension of Bs-2, Bs-4 and Bs-5 was diluted 100 times with sterilized water, and sprayed into three other *Aquilaria sinensis* except the control. Then *Aquilaria sinensis* was incubated for a month and replicons representing bacterial diversity (V3-V4 region) in leaves, stems and roots were sequenced. (**B**): The extracts of roots, stems and leaves of each group were tested by HPLC to determine the contents of agarotetrol, 6,7-dimethoxy-2-(2-phenylethyl)chromone and hydrocinnamic acid. (Standard 1: agarotetrol; Standard 2: 6,7-dimethoxy-2-(2-phenylethyl)chromone; Standard 4: hydrocinnamic acid). Values are expressed as mean ± SEM, * *p* < 0.05, and *** *p* < 0.0001 vs. control.

Because the roles of Agarwood saprophytic *Bacilli* were determined, we anticipated that these strains would have a positive effect on *Aquilaria sinensis*. Therefore, we selected strains Bs-2, Bs-4 and Bs-5 to inoculate them into the soil of *Aquilaria sinensis*. After 30 days, the *Bacillus* content of strains Bs-2, Bs-4 and Bs-5 in each part of the *Aquilaria sinensis* was tested by replicon sequencing (Figure 6A). However, the results showed that the percentage of *Bacillus* in the roots and stems decreased compared to the control. There was a difference between the root bacterial species group and the control. In the leaves, only the Bs-2 group was detected. The final experimental results indicated that the *Bacillus* content did not increase. At the same time, we also detected content changes of agarotetrol, 6,7-dimethoxy-2-(2-phenylethyl)chromone and hydrocinnamic acid in different parts of *Aquilaria sinensis* (Figure 6B). The results indicated that agarotetrol was only detected in the stem, and the content of agarotetrol was increased one month after inoculation with Bs-2 and Bs-5. 6,7-dimethoxy-2-(2-phenylethyl)chromone can be detected in the roots, stems and leaves of *Aquilaria sinensis*, and the content of 6,7-dimethoxy-2-(2-phenylethyl)chromone increased after inoculation with Bs-2 and Bs-5. In the leaves, only the content of 6,7-dimethoxy-2-(2-phenylethyl)chromone increased after inoculation with Bs-5. After inoculation of Bs-2 and Bs-5, the content of 3 components in stems all increased, and after inoculation of Bs-4, the content of 6,7-dimethoxy-2-(2-phenylethyl)chromone in stems and hydrocinnamic acid in leaves also increased.

## 3. Discussion

In this study, we found that the saprophytic *Bacillus* isolated from Agarwood can promote the release of its major components by degrading cellulose, while these strains had little effect on *Aquilaria sinensis*. This special mechanism explained the reason for the gradual release of fragments of Agarwood over the long-term; this effect has not been reported.

Indeed, there is a balanced confrontation between endophytic virulence factors and the host’s defense and immune system, and they coexist and co-evolve for a lifetime [25]. If the virulence factor released by the microorganism exceeds the resistance of the host’s defense system, the plant will show symptoms. Conversely, the microorganism will be restrained. Up to now, there are several endophytes that are deduced opportunistic pathogens, which can affect the host’s physiological state [26]. Here we detected *Bacillus* strains in *Aquilaria sinensis* through replicon sequencing, while these *Bacillus* strains displayed distinct functions as saprophytic bacteria to release major components of Agarwood. Although endophyte infection will not cause symptoms in living trees, it may change the plant hormones, which can induce the plant defensive enzymes and stimulate production of plant secondary metabolites or even new compounds such as oxidation products of octa decatrienoic acid (a-linolenic acid), octadecadienoic acid (linoleic acid) and oxophytodienoic acid [27,28]. It should also be noted that the cellulose in fresh plants and dead plants is different [29]. In living plants, endophytes may use carbon sources other than cellulose. Therefore, even though these *Bacillus* strains were detected in *Aquilaria sinensis*, they may not utilize cellulose until the death of the plant. This can also explain why the isolated *Bacillus* has no effect in *Aquilaria sinensis*, while it has significant effects on Agarwood.

Interestingly, we also used these endophytes for treatment of *Zanthoxylum bungeanum* Maxim, *Illicium verum* Hook. f, *Cinnamomum cassia* Presl, *Phellodendron chinense* Schneid, *Dalbergiaod orifera* T. Chen. However, our results indicated they were not as effective as against Agarwood. We deduce that this is because of their different contents and characteristics of cellulose. Cellulose is the main component of plant cell walls. The cellulose in different parts of the plant is different [30]. Among the medicinal materials, *Zanthoxylum bungeanum* Maxim and *Illicium verum* Hook. f are the peel and fruit of the plant *Zanthoxylum bungeanum* Maxim and *Illicium verum* Hook. f, respectively, suggesting that the cellulose content is not as high as Agarwood. Therefore, the slow degradations on *Zanthoxylum bungeanum* Maxim and *Illicium verum* Hook. f had no effect on the lease of their major components. Secondly, different antibacterial agents were contained in some of these medicinal materials. For example, the main active ingredient of *Phellodendron chinense* Schneid is berberine, which has a broad antibacterial effect [31,32]. The total alkaloids of *Zanthoxylum bungeanum* Maxim are reported to have similar effects [33,34]. Thus, bacteria may be inhibited in the presence of berberine or total alkaloids when degrading the cellulose of *Zanthoxylum bungeanum* Maxim. Third, the active components of different plants are extracellular products. Therefore, endophytes do not release more ingredients even though they can degrade the cell wall. 

Collectively, we demonstrated the role of saprophytic *Bacilli* of Agarwood as an accelerator in releasing the major components. We provided a promising method by spraying these *Bacilli* during the storage of Agarwood to ensure the effectiveness of this precious medicinal material.

## 4. Materials and Methods

### 4.1. Isolation of Saprophytic Microorganism

Samples of 10-year-old Agarwood were purchased from Hebei anguo Traditional Chinese medicine market. After washing the surface dust of Agarwood by sterilized water, the Agarwood was soaked in 70% ethanol for 1 min and rinsed five times in sterile distilled water, and then they were air-dried in an ultra-clean biosafety hood. To check the effectiveness of the sterilization procedures, the sterile distilled water used in the final rinse was plated onto PDA agar plate and incubated at 37 °C to confirm there was no contamination. The humus on the surface of the Agarwood was cut off by a sterilized blade in an ultra-clean table. Several 1 cm × 1 cm pieces in the center of the Agarwood was taken and placed on a PDA plate. The plate was then incubated in a 37 °C constant temperature incubator for 48 h. After optical colonies were obtained around the Agarwood piece, single colonies were transferred into a new medium, and this procedure was repeated three times to ensure the purity of the isolated microorganisms. Finally, the screened strains were preserved by the glycerol preservation method at −80 °C.

### 4.2. Agarwood Powder Fermentation

Agarwood pieces were smashed in a grinder and filtered through a 1 mm molecular sieve. Then, 0.3 g of Agarwood powder was mixed in sterilized saline for determination of degradation. Each 10 mL of the above mixture was added into two test tubes with stoppers, and then 10 µL of bacteria Bs-2 and Bs-4 respectively was added. The suspension was thoroughly mixed and incubated under shaking conditions (200 rpm, 37 °C). Samples were taken at 0 h, 24 h, and 48 h and filtered through a 0.22 µm filter membrane and determined by HPLC system (Shimazu, Kyoto, Japan) equipped with a C18 column and a SPD-20A UV detector at 252 nm. The column temperature was 30 °C and the mobile phase was composed of acetonitrile and 0.1% formic acid, and the detection was carried out by gradient elution at a flow rate of 0.7 mL/min. During the gradient elution process, the proportion of 0.1% formic acid solution in the mobile phase changed with time, and the settings were as follows: 0–5 min, 100–90%; 5–10 min, 90–85%; 10–25 min, 85–80%; 25–35 min, 80–70%; 35–50 min, 70–65%; 50–60 min, 65–55%; 60–70 min, 55–45%; 70–80 min, 45–35%; 80–90 min, 35–30%; 90–100 min, 30%; 100–120 min, 100%. The column was maintained at 30 °C. 

### 4.3. Cellulose Degradation

Cellulose degradation was conducted as follows. Strains were picked from the −80 °C refrigerator and transferred to sterilized tubes with LB medium, and cultured for 12 h under shaking conditions (200 rpm, 37 °C). The prepared CMC-Congo red medium was sterilized and poured onto the plate, and the bacterial suspension was dipped and placed on the center of the plate. Then the plate was cultured for 48 h at 37 °C. 

### 4.4. Cellulase Activity Detection

The enzyme activity of the seven strains was screened by the filter paper enzyme activity test. Then, 1 mL of the bacterial solution was put into a test tube, and 1 mL of citric acid-sodium citrate buffer solution at pH 4.5 and a 1 cm × 6 cm filter paper were added. Glucose detection was finished by the DNS method. Briefly, after preheating for 5 min at 50 °C, 3 mL DNS chromogenic solution was added for 1 h in a water bath and 10 min in a boiling water bath. The absorbance at 540 nm was measured after cooling. One unit of enzyme activity was defined as the amount of enzyme used to generate 1 mmol glucose in 1 min.

### 4.5. Degradation of Effective Components of Agarwood

One hundred microliters of standard solution (agarotetrol, 6,7-dimethoxy-2-(2-phenylethyl)chromone and 2-(2-phenethyl)chromone) of Agarwood (240 µg/mL) was separately put in a test tube with 10 mL of liquid LB medium and 10 µL of bacterial solution. The mixture was incubated under shaking conditions (200 rpm, 37 °C). Samples were taken at 0 h, 24 h and 48 h, and then filtered through a 0.22 µm membrane for HPLC analysis.

### 4.6. Effect of Saprophytic Bacteria on the Other Traditional Medicines

Each traditional medicine (Zanthoxylum bungeanum Maxim, Illicium verum Hook. f, Cinnamomum cassia Presl, Phellodendron chinense Schneid, *Dalbergiaod orifera* T. Chen) was smashed in a grinder and filtered through a 1 mm molecular sieve. Then, each medicine powder was added in sterilized saline for determination of degradation. Each 10 mL of the above mixture was added into two test tubes, and then 10 µL of bacteria Bs-2 and Bs-4 respectively was added. The suspension was thoroughly mixed and incubated under shaking conditions (200 rpm, 37 °C). Samples were taken at 0 h, 24 h, and 48 h and filtered through a 0.22 µm filter membrane for HPLC analysis.

The Dalbergiaod orifera T. Chen extract was determined by high performance liquid chromatography (HPLC) system (Shimazu, Kyoto, Japan) equipped with a C18 column and a SPD-20A UV detector at 270 nm. The column temperature was 30 °C and the mobile phase was methanol and 0.1% phosphoric acid in a volume ratio of 3:2, at a flow rate of 0.6 mL/min.

The extracts of Phellodendron chinense Schneid, Agarwood, Cinnamomum cassia Presl, Illicium verum Hook. f, and Zanthoxylum bungeanum Maxim were determined by HPLC system (Shimazu, Kyoto, Japan) equipped with a C18 column and a SPD-20A UV detector at 252 nm. The column temperature was 30 °C and the mobile phase was composed of acetonitrile and 0.1% formic acid, and the detection was carried out by gradient elution at a flow rate of 0.7 mL/min. During the gradient elution process, the proportion of 0.1% formic acid solution in the mobile phase changed with time, and the settings were as follows: 0–5 min, 100–90%; 5–10 min, 90–85%; 10–25 min, 85–80%; 25–35 min, 80–70%; 35–50 min, 70–65%; 50–60 min, 65–55%; 60–70 min, 55–45%; 70–80 min, 45–35%; 80–90 min, 35–30%; 90–100 min, 30%; 100–120 min, 100%. The column was maintained at 30 °C.

### 4.7. Replicon Sequencing in Aquilaria sinensis

A certain amount of vermiculite was sterilized and distributed to flower pots after cooling. Four *Aquilaria sinensis* were transplanted into flower pots. The Bs-2, Bs-4 and Bs-5 strains were taken respectively in sterilized LB liquid medium and incubated for 12 h under shaking conditions (200 rpm, 37 °C). One milliliter of Bs-2, Bs-4 and Bs-5 strains was diluted 100 times with sterilized water, and sprayed into three other *Aquilaria sinensis* except the control. Then, *Aquilaria sinensis* was incubated in an incubator for a month under optimal conditions (25 °C, 75% of humidity, 50% of light). After 1 month, replicons representing bacterial diversity (V3-V4 region) in leaves, stems and roots were sequenced. DNA sequencing and data analysis were carried out in Hanyu Biotech Co. Ltd., Shanghai.

## Figures and Tables

**Table 1:** Determination of cellulase activity.

Strains	Bs-1	Bs-2	Bs-3	Bs-4	Bs-5	Bs-6	Bs-7
FPA (U/mL)	0.15	0.20	0.11	0.22	0.14	0.12	0.12

## Data Availability

Original data of this work are available by email request to the corresponding author.

## Sample Availability

Samples and strains using this work are available from the authors.

## Appendix A

### Appendix A.3. Replicon Sequencing in Aquilaria sinensis

**Table A1:** Saprophytic *Bacillus* effected on the root of *Aquilaria sinensis*.

Content	Control	Bs-2	Bs-4	Bs-5
*Bacillus* in root(%)	0.0037	0.0006	0.0012	0.0013
*Bacillus* in root(%)	0.0031	0.0021	0.0014	0.0011
*Bacillus* in root(%)	0.0035	0.0011	0.0012	0.0023

**Table A2:** Saprophytic *Bacillus* effected on the stem of *Aquilaria sinensis*.

Content	Control	Bs-2	Bs-4	Bs-5
*Bacillus* in stem(%)	0.0039	0.0029	0.0026	0.0285
*Bacillus* in stem(%)	0.0085	0.0000	0.0004	0.0009
*Bacillus* in stem(%)	0.0000	0.0000	0.0000	0.0057

**Table A3:** Saprophytic *Bacillus* effected on the leaf of *Aquilaria sinensis*.

Content	Control	Bs-2	Bs-4	Bs-5
*Bacillus* in leaf(%)	0.0000	0.0001	0.0000	0.0000
*Bacillus* in leaf(%)	0.0000	0.0003	0.0000	0.0000
*Bacillus* in leaf(%)	0.0000	0.0050	0.0000	0.0000
